# Case report: Meningitis: a cause of reversible cerebral vasoconstriction syndrome?

**DOI:** 10.3389/fneur.2023.1143215

**Published:** 2023-07-20

**Authors:** Fida Oukhai, Valérie Domigo, Joseph Benzakoun, Michel Wolff, Anne Ducros, Jean-Louis Mas, David Calvet

**Affiliations:** ^1^Neurology Department, Groupe Hospitalier Universitaire (GHU) Paris Psychiatrie et Neurosciences, Sainte-Anne Hospital, Université de Paris, Federation Hospitalo-Universitaire (FHU) NeuroVasc, Paris, France; ^2^INSERM 1266, Institut de Psychiatrie et Neurosciences de Paris, Paris, France; ^3^Radiology Department, Groupe Hospitalier Universitaire (GHU) Paris Psychiatrie et Neurosciences, Sainte-Anne Hospital, Paris, France; ^4^Neuro Intensive Care Unit, Groupe Hospitalier Universitaire (GHU) Paris Psychiatrie et Neurosciences, Sainte-Anne Hospital, Paris, France; ^5^Neurology Department, Gui de Chauliac Hospital, Centre Hospitalier Universitaire (CHU) de Montpellier, Montpellier, France

**Keywords:** RCVS, meningitis, thunderclap headache, reversible cerebral vasoconstriction syndrome, CSF inflammation

## Abstract

**Introduction:**

Reversible cerebral vasoconstriction syndrome (RCVS) is characterized by thunderclap headache and reversible cerebral arteries vasoconstriction. The pathophysiology remains unclear, but many triggers were reported.

**Case reports:**

We reported two cases of patients with meningitis who developed RCVS confirmed by brain imaging. They presented clinical and CSF features of meningitis that are suspected to be infectious, but no agent was identified. Headache and artery irregularities were resolved with the improvement of CSF.

**Conclusion:**

These cases suggest that in the context of meningitis, modification or atypical headaches should lead to brain imaging to rule out RCVS. We hypothesized that CSF inflammation may trigger cerebral arteries vasoconstriction.

## Introduction

Reversible cerebral vasoconstriction syndrome (RCVS) is a clinical and radiological syndrome characterized by severe headaches, often of the thunderclap type, with reversible vasospasm of intracranial arteries (1). Although the pathogenesis is not well understood, various precipitants have been identified such as exposure to drugs, vasoactive medication, pregnancy, and postpartum (2).

In this study, we reported two cases of RCVS associated with meningitis.

## Case report

### Case 1

A 62-year-old woman awoke with severe cervical, dorsal, and lumbar pain and a severe headache. She had a past medical history of chronic depression treated by duloxetine initiated several months ago, active cigarette smoking, and transient global amnesia. A few hours later, her headache acutely worsened (Visual Analog Scale for Pain at 9/10 in a few seconds). The headache was aggravated by neck movements, with overall seven episodes of thunderclap headaches in 24 h. Opioid analgesics were prescribed without improvement of pain. After 3 days, she presented to the emergency department because of persistent headache, a temperature of 38.2°C, and neck stiffness. Laboratory findings showed a biological inflammatory syndrome with serum white blood cells at 10,660 cells/μl (neutrophils 8,310 cells/μl) and a C-reactive protein of 85 mg/L. The cerebrospinal fluid contained 2,600 leukocytes/mm^3^ with 68% neutrophils, a protein concentration of 148 mg/dL, and a glucose concentration of 0.6 mmol/L (blood glucose: 5.8 mmol/L). The gram stain was negative, but a diagnosis of bacterial meningitis was suspected. Blood and CSF cultures as well as a multiplex PCR were negative. A brain computed tomography (CT) was normal, but the CT angiography showed multiple intracranial stenosis: distal branches of the middle cerebral arteries and a distal segment of the anterior cerebral artery. The diagnosis of RCVS was suspected, and the patient was transferred to our acute stroke unit. The patient received a combination of intravenous cefotaxime (20 mg/kg/day), amoxicillin (200 mg/kg/day), and dexamethasone. Intravenous nimodipine was started to treat RCVS.

**Figure 1:**
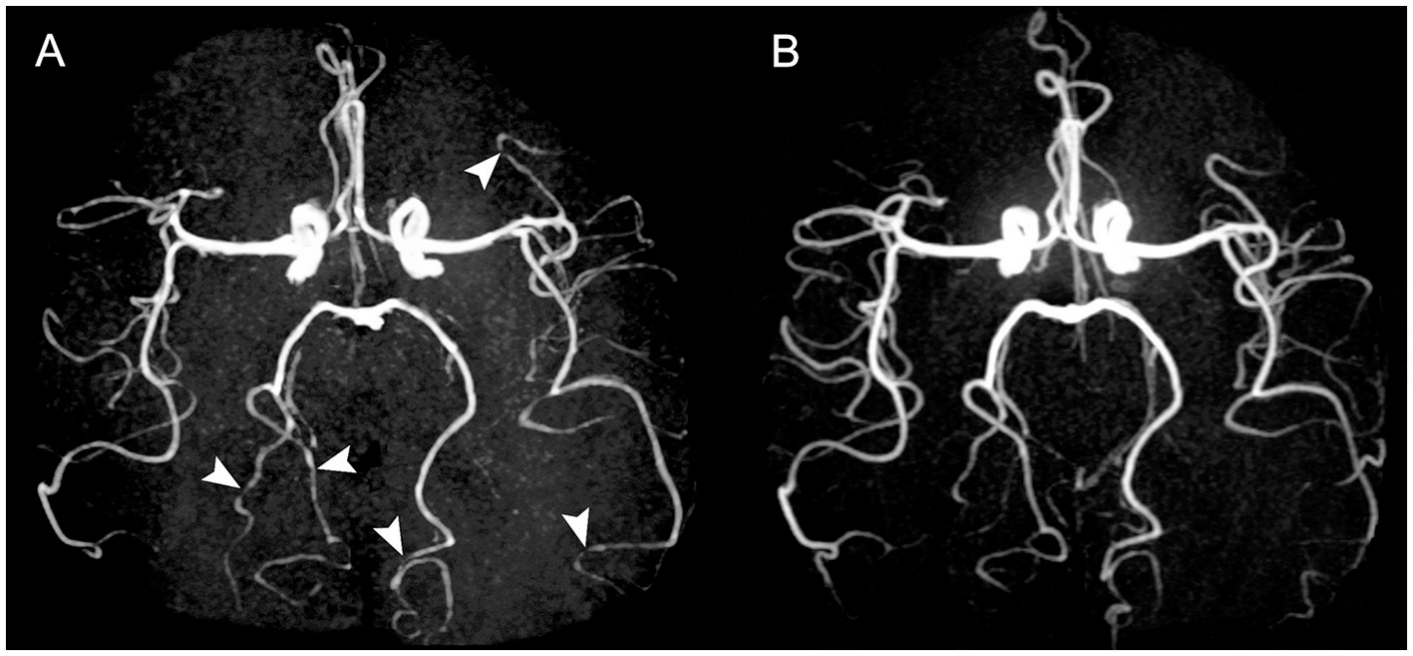
Time-of-flight MR angiographies of patient 1. Initial examination **(A)** shows multiple stenoses (arrowheads) involving P3 segment of right and left posterior cerebral artery, and M3 and M4 segments of left middle cerebral artery. Control MRA at 5 months **(B)** shows a complete resolution of these stenoses.

Brain MRI confirmed the absence of brain lesions but the presence of several bilateral arterial irregularities on MR angiography (3D time of flight) (Figure 1). Cervical echo-Doppler ultrasound identified signs of old bilateral carotid dissection without recent hematoma. Transcranial echo-Doppler ultrasound confirmed multiple irregularities of brain arteries. MRI with fat-saturated T1-weighted sequences found no recent wall hematoma of carotid arteries supporting the diagnosis of sequelae.

She did not report further thunderclap headaches. She received 2 days of cefotaxime and 23 days of amoxicillin. We switched to oral nimodipine (60 mg every 4 h) on the third day for a total duration of 1 month. After 5 days, CSF contained 46 leukocytes/mm^3^ with 73% neutrophils, a glucose level of 1.43 mmol/l, and protein at 148 mg/dL. At 2 months, neurological examination was normal, CSF contained 9 cells/mm^3^, and the protein level was 42 mg/dL. MRA and transcranial echo-Doppler ultrasound found very moderate distal stenosis. At 5 months, CSF leukocyte count was 3 cells/mm^3^ with a protein level of 35 mg/dL, and MRI found a resolution of cerebral artery stenosis.

### Case 2

A 47-year-old woman had a sudden onset of severe headache with vomiting and nausea. She had a medical history of penicillin allergy, active cigarette smoking, and depression treated with fluoxetine for many years. Brain CT was normal. Headaches remained severe, and the patient stayed at home. A week later, she developed a fever and visual hallucinations. Initial examination at the emergency department found a confused patient with a fever of 39.3° and gait disturbance. Blood pressure was 170/90 mm Hg in a patient without any history of hypertension. Laboratory findings showed a biological inflammatory syndrome with serum white blood cells at 11 × 10^3^/μl (neutrophils 8.280 × 10^3^ cells/μl) and 11 C-reactive protein. CSF contained 193 leukocytes/mm^3^ with 86% neutrophils, a protein concentration of 210 mg/dL, and a glucose concentration of 3.1 mmol/L (blood glucose: 7.7 mmol/L). CSF gram stain was negative. A presumed diagnosis of bacterial meningitis was made, and she received empirical antibiotic therapy with cefotaxime and amoxicillin combined with aciclovir pending microbiological testing. Blood and CSF cultures as well as a multiplex PCR were negative. Thoracoabdominal CT did not show any infection. Brain MRI revealed bilateral T2-weighted FLAIR hyper-intensities in posterior areas, and initial 3D time of flight angiography did not identify any stenosis. A diagnosis of posterior reversible encephalopathy syndrome was suspected. Antibiotics were stopped after 2 days as she no longer had a fever and biological inflammatory syndrome, and CSF results were negative. The patient was transferred to our neurology referral center.

**Figure 2:**
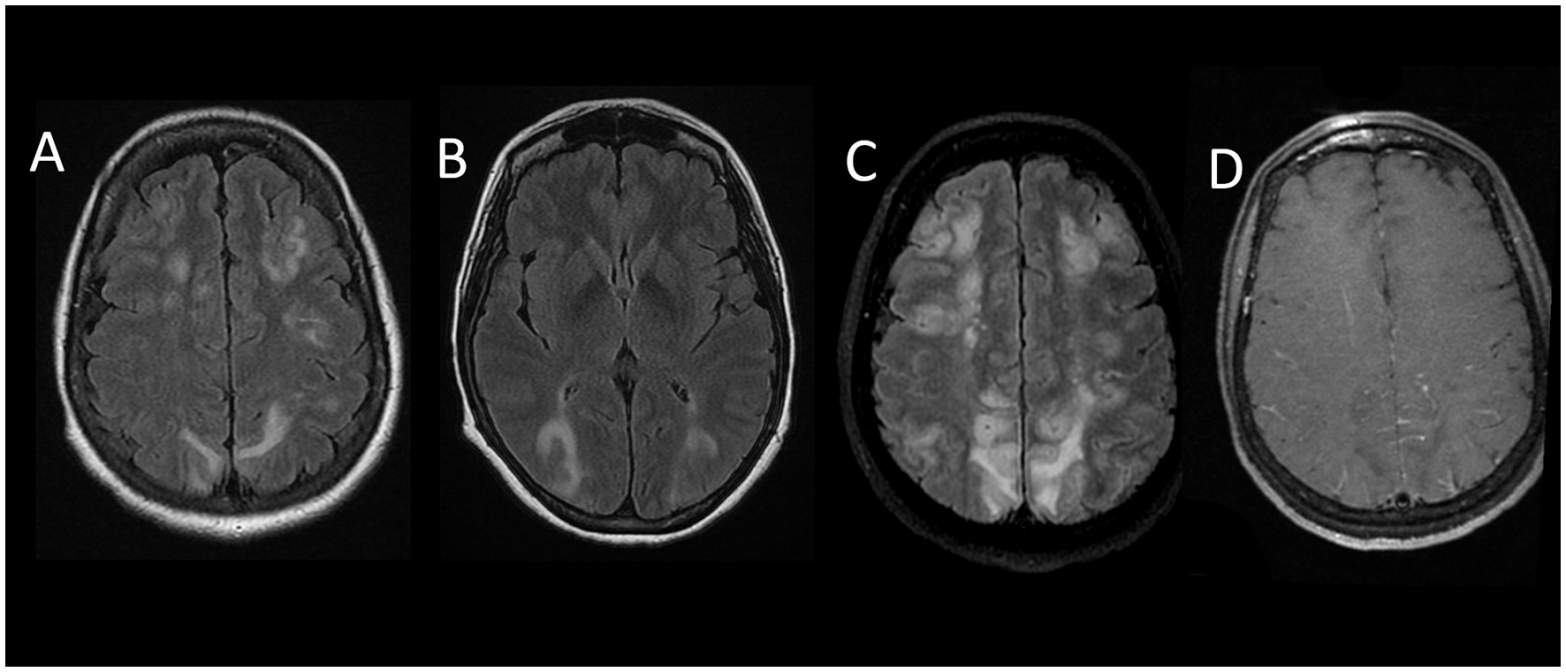
Initial brain MRI of patient 2 **(A, B)** shows multiple bilateral confluent hyperintense lesions involving the subcortical white matter and diffuse cerebral edema (FLAIR sequence, axial view). MRI after clinical worsening (a week later) **(C)**: extension of FLAIR hyper-intensities in parietal and frontal lobes associated with gadolinium enhancement **(D)**.

**Figure 3:**
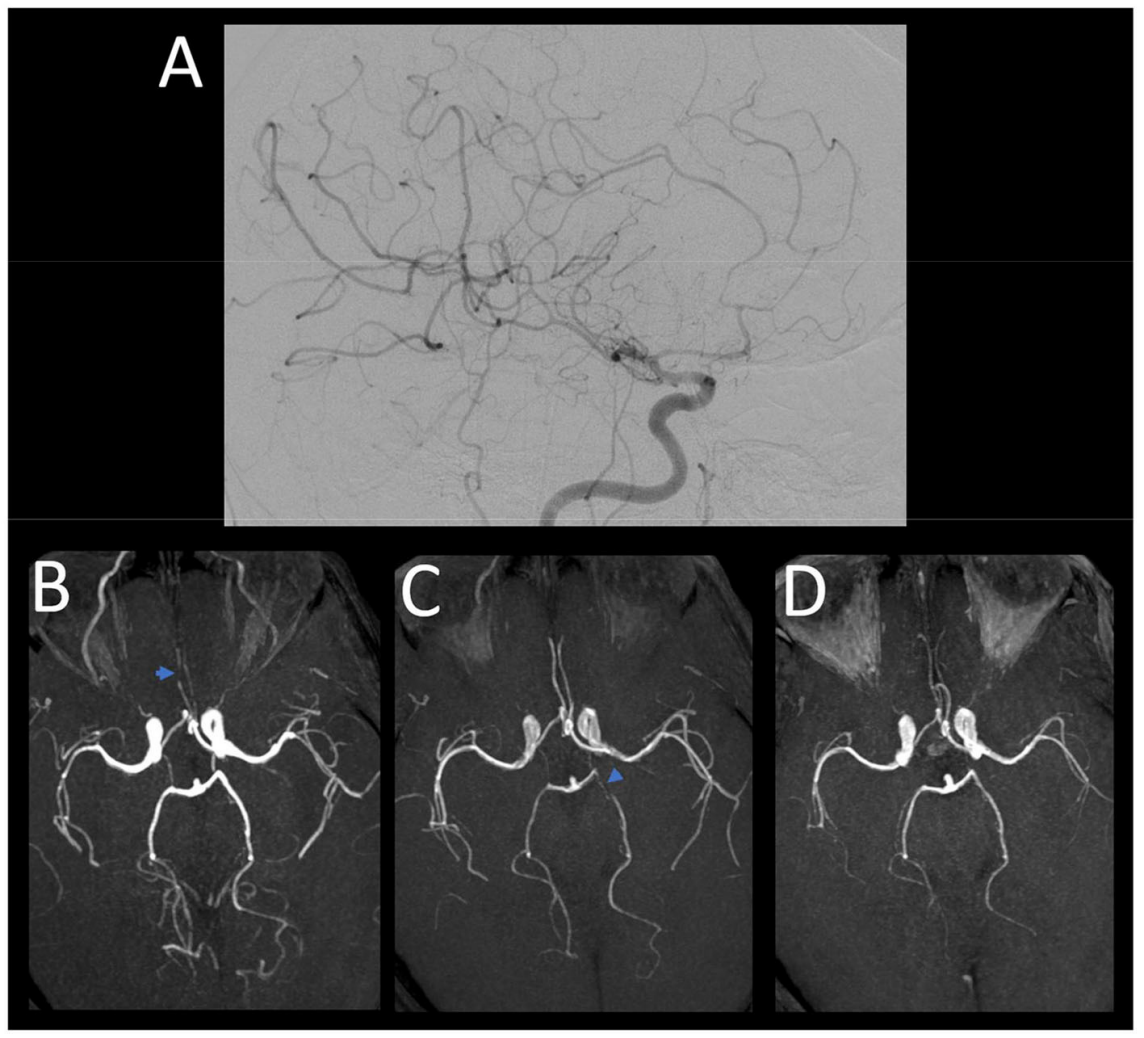
Digital subtraction angiography in patient 2 **(A)** performed 2 days after admission shows narrowing of proximal segments involving middle (M1) and anterior (A1) arteries. 3D time of flight angiography in the same patient a day after **(B)** shows stenoses of anterior cerebral artery (A2-A3). A week later control **(C)** shows proximal stenosis of left posterior cerebral artery. Resolution of cerebral artery stenoses at 6-month control **(D)**.

The day after admission, she presented a worsening of her condition with two probable focal seizures revealed by impaired awareness and chewing movements (the longest-lasting period was 30 min). Examination found paralysis of the right upper limb and left lower limb and paresis of the right lower limb and right hypoesthesia. MRI revealed an extension of FLAIR and DWI hyper-intensities in the occipital and parietal lobes and the cerebellum with cortical laminar necrosis of the left precentral gyrus and occipital lobes (Figure 2). Sulcal hyper-intensities associated with gadolinium enhancement were observed. The 3D time of flight angiography was normal, but transcranial Doppler ultrasonography showed multiple focal increased velocities on brain arteries. Digital subtraction angiography showed narrowing of proximal segments involving middle, anterior, and posterior cerebral arteries (Figure 3). The diagnosis of RCVS was suspected, and intravenous nimodipine (1 mg/h) was started. Arterial lesions were also identified on 3D time of flight angiography performed the day after digital subtraction angiography. CSF on day 5 showed 290 leukocytes/mm^3^ with a protein level of 102 mg/dL. Neurological outcomes were favorable with progressive improvement of consciousness during 2 weeks after epileptic seizures and motor deficit during 3 weeks. After 2 weeks (1 month after onset), MR angiography and transcranial Doppler ultrasound were subnormal with only one moderate proximal posterior cerebral artery stenosis, and CSF was normal. Unfortunately, 1 year after the event, she had sequels of moderate right upper hand paresis.

## Discussion

These two patients developed RCVS in the context of meningitis. Brain imaging can show contrast leakage into subarachnoid space on contrast-enhanced FLAIR imaging in approximately half of the patients with RCVS, supporting blood–brain barrier breakdown as an important physiopathological event (3). We hypothesized that inflammation of CSF during meningitis can be a trigger of endothelial dysfunction which is suspected to play a role in the pathophysiology of RCVS (4).

CSF abnormalities were reported in some series of patients with RCVS (1) but remained moderate and did not exceed 35 white blood cells and 101 mg/dL of protein level (5). Only two cases of meningitis associated with RCVS have been previously reported (6, 7). A woman developed RCVS in the context of postpartum and sumatriptan use, which are known to be triggers of this condition (6). She also had clinical and biological features of infectious meningitis without an identified infectious agent. Another 23-year-old woman had RCVS and abnormal CSF attributed to recurrent aseptic meningitis (7). Interestingly, in both our cases, infectious meningitis was strongly suspected, but no infectious agent was identified despite a detailed microbiological work-up including multiplex PCR. Thus, it cannot be excluded that RCVS itself may favor inflammatory meningitis.

Our two patients were also treated with specific serotonin reuptake inhibitors. These drugs are known to be precipitants of RCVS (1, 8), but the treatment had been started a long time before admission without any recent modification. Both patients had a history of active cigarette smoking but had no cannabis or nicotine patch use. In addition, a dissection also known to be associated with RCVS (9) was discussed in our first case but cervical echo-Doppler ultrasound and MRI ruled out any recent dissection. Regarding our second case, posterior reversible encephalopathy syndrome was associated with RCVS, but it is accepted that these two entities often coexist and have overlapping pathophysiology (10). We consider that CSF inflammation may have triggered both posterior reversible encephalopathy syndrome and RCVS in our second case.

These two cases suggest that, in the context of meningitis, modification or atypical headaches, in particular the occurrence of thunderclap headaches, should lead to brain imaging and cerebral arterial work-up to rule out RCVS.

## Clinical implications

- In the context of meningitis, thunderclap headaches should lead to cerebral arterial work-up to rule out RCVS.
- CSF inflammation may trigger reversible vasoconstriction of cerebral arteries.

## Data availability statement

The original contributions presented in the study are included in the article/supplementary material, further inquiries can be directed to the corresponding author.

## Ethics statement

Written informed consent was obtained from the individual(s) for the publication of any potentially identifiable images or data included in this article.

## Author contributions

FO wrote the first draft of the paper. VD was the physician of the two patients. JB was the radiologist and MW was the referring infectiologist in these cases. DC had the final responsibility for manuscript content. All authors made substantial contributions to the conception of the study, acquisition of data or analysis, and critical revision of the article.
